# Fine-tuning levels of heterologous gene expression in plants by orthogonal variation of the untranslated regions of a nonreplicating transient expression system

**DOI:** 10.1111/pbi.12175

**Published:** 2014-03-12

**Authors:** Yulia A Meshcheriakova, Pooja Saxena, George P Lomonossoff

**Affiliations:** Department of Biological Chemistry, John Innes CentreNorwich, UK

**Keywords:** Translational efficiency, viral 3′UTR, CPMV-*HT*, Y-shaped secondary structure, pEAQ vectors

## Abstract

A transient expression system based on a deleted version of Cowpea mosaic virus (CPMV) RNA-2, termed CPMV-*HT,* in which the sequence to be expressed is positioned between a modified 5′ UTR and the 3′ UTR has been successfully used for the plant-based expression of a wide range of proteins, including heteromultimeric complexes. While previous work has demonstrated that alterations to the sequence of the 5′ UTR can dramatically influence expression levels, the role of the 3′ UTR in enhancing expression has not been determined. In this work, we have examined the effect of different mutations in the 3′UTR of CPMV RNA-2 on expression levels using the reporter protein GFP encoded by the expression vector, pEAQexpress-HT-GFP. The results showed that the presence of a 3′ UTR in the CPMV-*HT* system is important for achieving maximal expression levels. Removal of the entire 3′ UTR reduced expression to approximately 30% of that obtained in its presence. It was found that the Y-shaped secondary structure formed by nucleotides 125–165 of the 3′ UTR plays a key role in its function; mutations that disrupt this Y-shaped structure have an effect equivalent to the deletion of the entire 3′ UTR. Our results suggest that the Y-shaped secondary structure acts by enhancing mRNA accumulation rather than by having a direct effect on RNA translation. The work described in this paper shows that the 5′ and 3′ UTRs in CPMV-*HT* act orthogonally and that mutations introduced into them allow fine modulation of protein expression levels.

## Introduction

**Figure 1:**
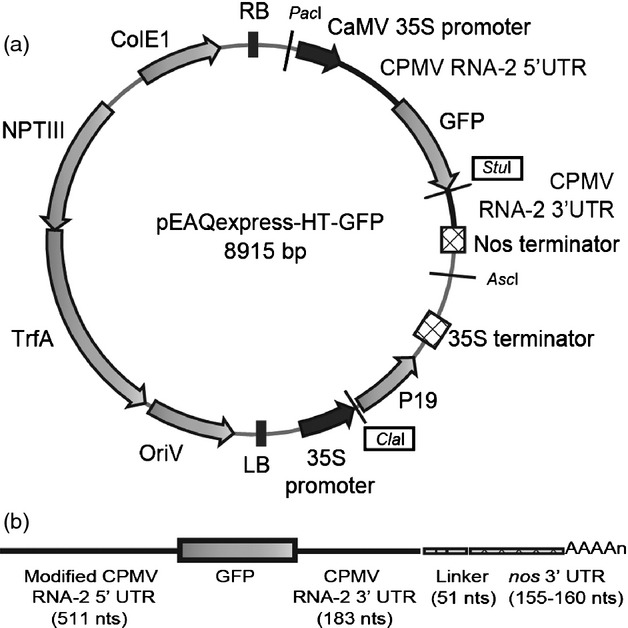
Schematic representation of construct used for GFP expression (a) Plasmid diagram of pEAQexpress-HT-GFP. Dark arrows represent promoters, light arrows represent open reading frames, checked boxes represent terminators, and solid lines represent the UTRs from CPMVRNA-2 system. (b) Schematic representation of the GFP-specific mRNA transcribed from construct pEAQexpress-HT-GFP. Solid lines denote UTRs from CPMV RNA-2, the lined box denotes the 51 nucleotide linker, and the checked box denotes the 3′ UTR from the *nos* gene.

The Cowpea mosaic virus hypertranslatable (CPMV-*HT*) system and its associated pEAQ vectors have become a popular system for high-level transient expression of heterologous proteins in plants via agro-infiltration (for a recent review, see Peyret and Lomonossoff, 2013). This system involves positioning the target gene between a modified 5′UTR, from which upstream AUG codons have been deleted, and a wild-type 3′UTR from CPMV RNA-2, to create a ‘hypertranslatable’ (*HT*) cassette within the T-DNA region of a binary vector. Transcription from the *HT* cassette is driven by a Cauliflower mosaic virus (CaMV) 35S promoter and is terminated by a nopaline synthase (*nos*) terminator. This results in the production of an mRNA in which the inserted gene is flanked at its 5′ end by the modified CPMV 5′ UTR and at its 3′ end by the 3′ UTR from CPMV RNA-2, followed by a linker sequence and the 3′ UTR from the *nos* gene (Figure 1b).

While the importance of the modified 5′ UTR in achieving high-level expression using the CPMV-*HT* system is well documented (Peyret and Lomonossoff, 2013; Sainsbury and Lomonossoff, 2008; Sainsbury *et al.*, 2010; Thuenemann *et al.*, 2013), the role of the 3′ UTR, if any, is unknown. The sequence of the 3′ UTR was originally included in CPMV-based expression plasmids to allow replication of RNA transcripts by the CPMV RNA-1-encoded polymerase (Cañizares *et al*., 2006), an ability not required in the CPMV-*HT* system. However, the sequence was retained on the grounds that many plant viral mRNAs contain a cap-independent translation element (3′-CITE) within the 3′UTR (for a review, see Miller *et al.*, 2007). These elements can be transplanted onto heterologous open reading frames and have been shown to promote their translation. Thus, it was rationalized that the sequence of the 3′ UTR of CPMV RNA-2 may assist high-level expression from RNAs transcribed from *HT* cassettes. In this paper, we show that this is, indeed, the case and that the enhancing effect operates by increasing mRNA accumulation. Furthermore, we show that the contributions of the 5′ and 3′ UTRs towards enhancing expression act orthogonally, and constructs directing varying levels of expression can be obtained by mixing and matching 5′ and 3′ UTRs.

## Results

### Comparison of GFP expression levels in the presence and in the absence of the 3′ UTR of CPMV RNA-2

The potential role of the 3′ UTR of CPMV RNA-2 in enhancing expression levels from pEAQ plasmids (Sainsbury *et al.*, 2009) was investigated using the construct pEAQexpress-HT-GFP (Figure 1a). This construct encodes the sequence of GFP within a CPMV-*HT* cassette and gives high-level expression of GFP when agro-infiltrated into 3-week-old *Nicotiana benthamiana* plants (Sainsbury *et al.*, 2009). In common with other pEAQ-based constructs, transcripts from the CPMV-*HT* cassette are anticipated to consist of the modified CPMV RNA-2 5′ UTR, followed by the structural gene (in this case *gfp*) and a chimaeric 3′ UTR (Figure 1b). The latter consists of 183 nucleotides corresponding to the 3′ UTR of CPMV RNA-2, a 51-nucleotide linker consisting of 18As derived from the poly(A) tail of RNA-2 plus a polylinker and, finally, 155-160 nucleotides (depending on the precise site of termination; Bevan *et al.*, 1983) from the 3′ UTR of the *nos* gene from pBINPLUS (van Engelen *et al.*, 1995; Figure S1). The transcripts are also expected to be capped at their 5′ end and polyadenylated at their 3′ end.

**Figure 2:**
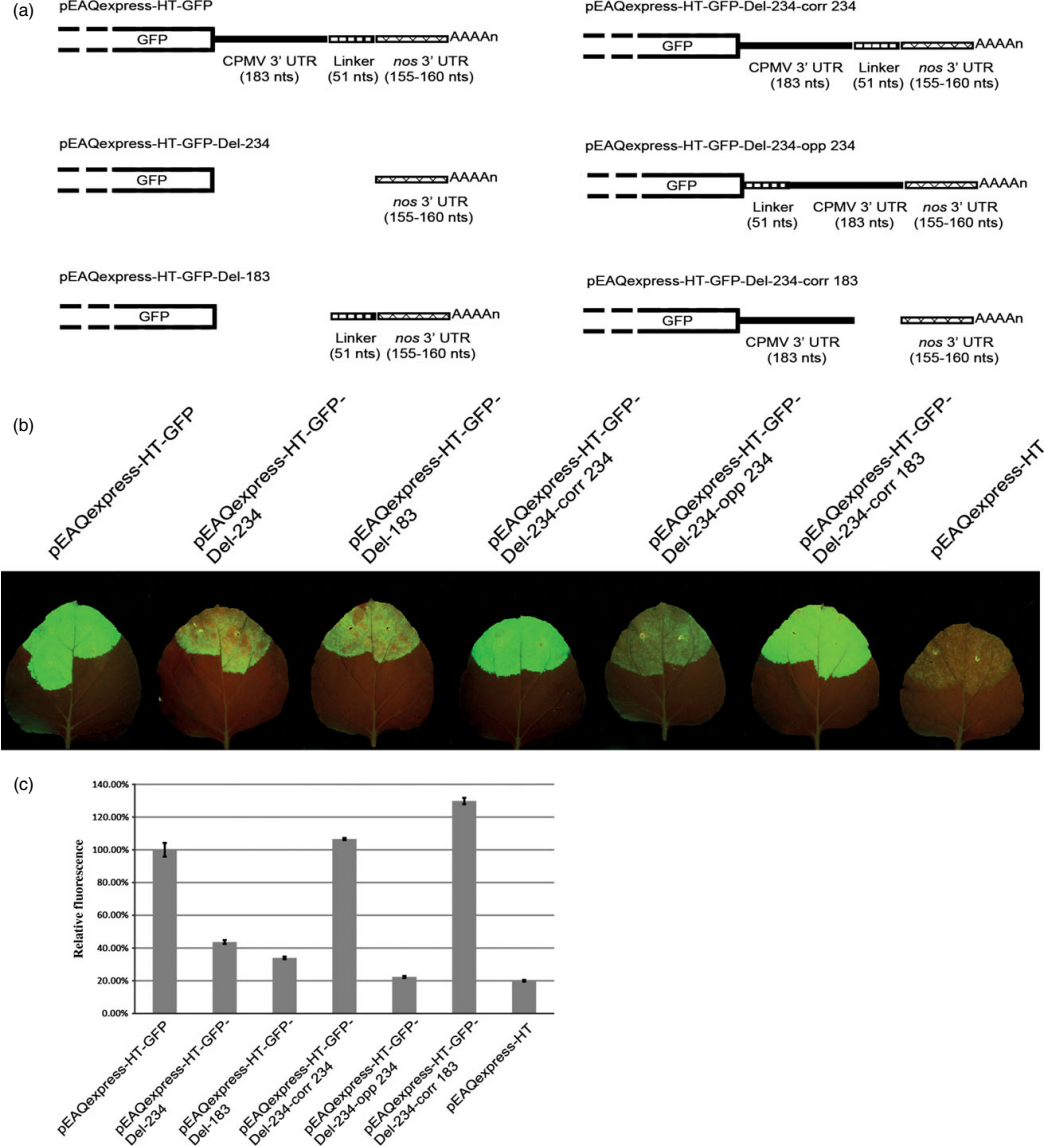
Analysis of the role of the 3′ UTR in achieving high-level expression. (a) Schematic representation of the series of mutants made upon deletion of different sections within the 3′ UTR of pEAQexpress-HT-GFP. (b) Leaves infiltrated with the various constructs (as indicated), photographed at 6 dpi under UV light. (c) Relative GFP expression levels based on spectrofluorometric analysis of extracts from leaves harvested at 6 dpi. Values indicate relative expression levels averaged from three technical replicates ± standard error.

To determine whether the sequences from the 3′ UTR of CPMV RNA-2 and the linker have any role in enhancing expression from the pEAQ vectors, either a 234-nucleotide sequence encompassing the entire 183-nucleotide RNA-2-derived 3′ UTR sequence plus the 51 nucleotide linker or just the RNA-2-specific 183 nucleotides were deleted from pEAQexpress-HT-GFP to give plasmids pEAQexpress-HT-GFP-Del-234 and pEAQexpress-HT-GFP-Del-183, respectively (Figure 2a). In pEAQexpress-HT-GFP-Del-234, the 3′ UTR from the *nos* gene is immediately adjacent to the sequence encoding GFP, while in pEAQexpressGFP-Del-183, it is separated by the 51-nucleotide linker. As controls, the 234 nucleotides deleted from pEAQexpress-HT-GFP-Del-234 were re-inserted in either the correct (pEAQexpress-HT-GFP-Del-234-corr 234) or opposite orientation (pEAQexpress-HT-GFP-Del-234-opp 234), and the 183-nucleotide sequence corresponding to the CPMV RNA-2 3′ UTR was inserted in the correct orientation (pEAQexpress-HT-GFP-Del-234-corr 183) using the unique *StuI* site of pEAQexpress-HT-GFP-Del-234 (Figure 2a). Following transformation into *Agrobacterium tumefaciens* and infiltration of *N. benthamiana* leaves, the level of GFP expression was assessed visually under UV light (Figure 2b), and the accumulation of GFP was quantified by spectrofluorometric analysis of the protein extracts of agro-infiltrated leaves. Elimination of the 234-nucleotide sequence encompassing both the CPMV RNA-2 3′ UTR and the linker or just the 183 nucleotides corresponding to the 3′ UTR resulted in a decrease in GFP expression levels to 43.64% and 33.94%, respectively, of that obtained with the original pEAQexpress-HT-GFP construct (Figure 2c). Re-insertion of the 234-nucleotide fragment in the correct but not the reverse orientation fully restored GFP expression; re-insertion of just the 183-nucleotide CPMV-specific region in the correct orientation was equally effective. The relative levels of fluorescence were precisely correlated with the levels of GFP detected on Coomassie blue-stained SDS-polyacrylamide gels (data not shown) consistent with previous results (Sainsbury and Lomonossoff, 2008; Sainsbury *et al.*, 2009). These results indicate that the presence of the 3′ UTR of CPMV RNA-2 in the correct orientation boosts expression of GFP up to threefold compared with the levels obtained when just the *nos* 3′ UTR is present.

### A secondary structure motif within the 3′ UTR of CPMV RNA-2 is important for enhancing expression

**Figure 3:**
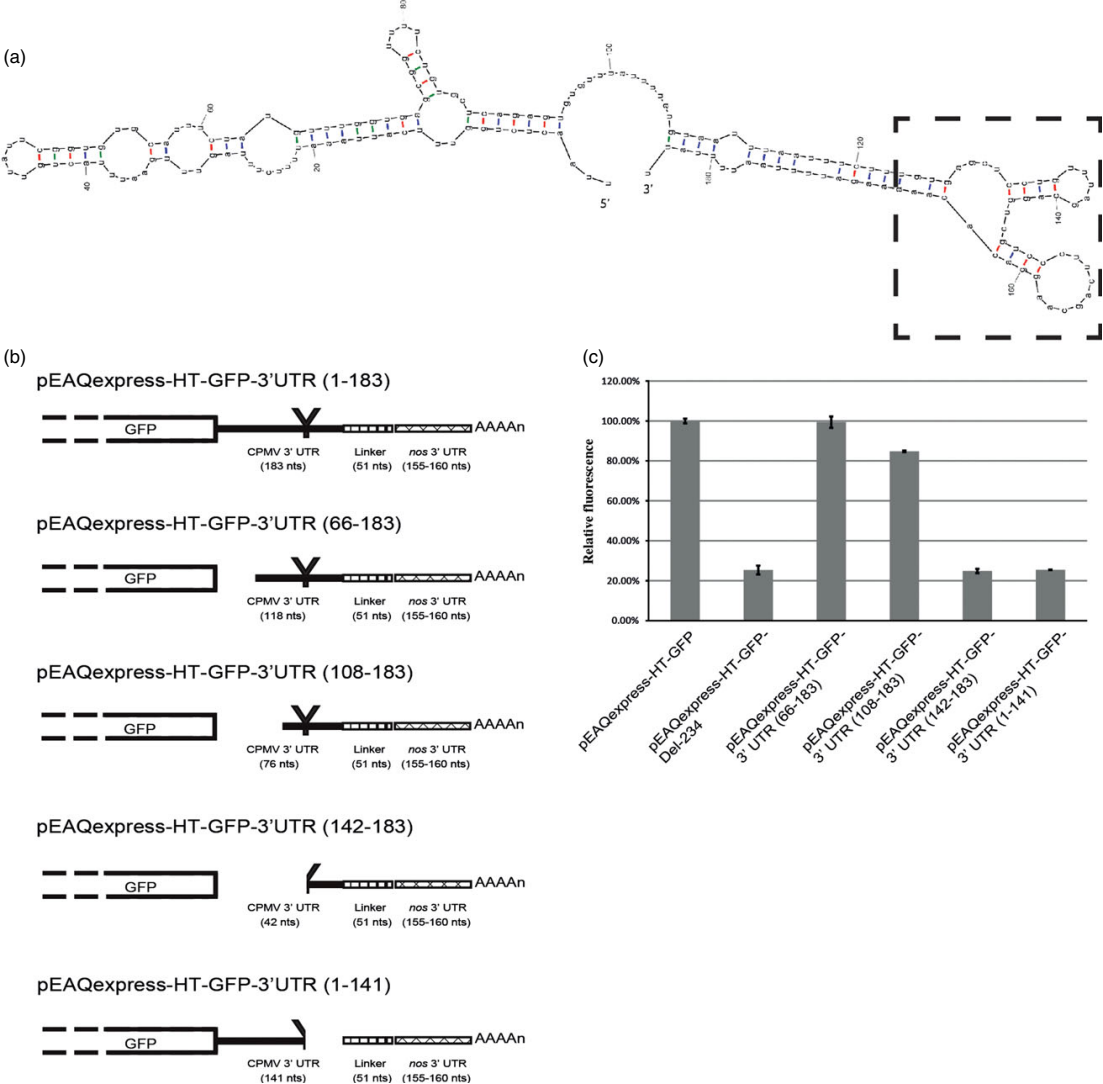
Deletion mutagenesis of the 3′ UTR. (a) Predicted secondary structure of the 3′UTR of CPMV RNA-2. The Y-shaped loop has been highlighted. (b) Schematic representation of the series of mutants constructed upon deletion of different sections within the 3′ UTR of CPMV RNA-2. (c) Relative GFP expression levels based on spectrofluorometric analysis of extracts from leaves harvested at 6 dpi. Values indicate relative expression levels averaged from three technical replicates ± standard error.

To define more precisely the region(s) of the 3′ UTR involved in enhancing expression levels, a series of deletion mutants based on pEAQexpress-HT-GFP was constructed (Figure 3b). Deletion of 65 and 107 nucleotides from the region of 3′ UTR immediately downstream of the GFP stop codon, resulting in plasmids pEAQexpress-HT-GFP-3′UTR(66-183) and pEAQexpress-HT-GFP-3′UTR(108-183), had only a limited effect on reducing expression levels, whereas deletion of 141 nucleotides in pEAQexpress-HT-GFP-3′UTR(142-183) reduced the expression level to that of pEAQexpress-HT-GFP-Del-234, which lacks the entire 3′ UTR (Figure 3c). This suggests that there is a region downstream of nucleotide 107 of the 3′ UTR of CPMV RNA-2 that is critical for maintaining high levels of expression from the pEAQ vectors, although upstream sequences may also play some role. To verify this, 93 nucleotides, including the 3′ terminal 42 nucleotides of the 3′ UTR plus the 51 nucleotides downstream, were deleted to give pEAQexpress-HT-GFP-3′UTR(1-141) (Figure 3b). Again, expression levels from this construct were equivalent to those obtained with pEAQexpress-HT-GFP-Del-234 (Figure 3c).

**Figure 4:**
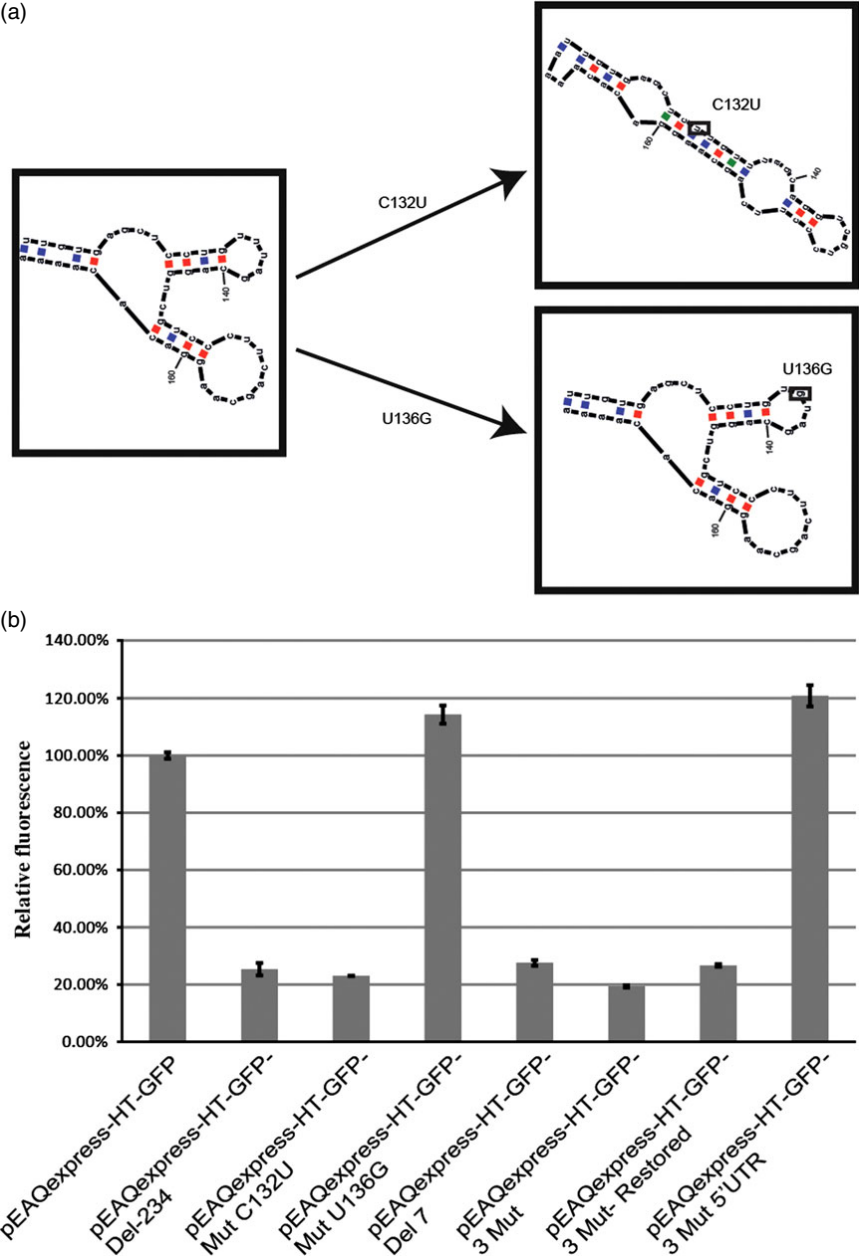
Importance of secondary structure in the 3′ UTR. (a) Secondary structures predicted for 3′ UTR point mutants C132U and U136G. (b) Relative GFP expression levels based on spectrofluorometric analysis of extracts from leaves harvested at 6 dpi. Values indicate relative expression levels averaged from three technical replicates ± standard error.

Secondary structure predictions of the 3′ UTR of CPMV RNA-2 have indicated that the region between nucleotides 125 and 165 can be folded into a stable Y-shaped stem-loop structure (Eggen *et al.*, 1989; Rohll *et al.*, 1993); (Figure 3a). This structure was subsequently shown to be important for efficient accumulation of RNA-2 during replication in protoplasts (Rohll *et al.*, 1993). As the deletions in pEAQexpress-HT-GFP-3′UTR(142-183) and pEAQexpress-HT-GFP-3′UTR(1-141) extend into this Y-shaped structure while those in pEAQexpress-HT-GFP-3′UTR(66-183) and pEAQexpress-HT-GFP-3′UTR(108-183) do not, it is plausible that the differential expression levels obtained relate to the presence or absence of the intact Y-shaped structure. To investigate this possibility, point mutations were introduced into pEAQexpress-HT-GFP, which were predicted either to disrupt the Y-shaped structure (C132U) or to leave it intact (U136G) (Figure 4a). Assessment of the GFP expression levels obtained with the plasmids harbouring these two mutations (pEAQexpress-HT-GFP-MutC132U and pEAQexpress-HT-GFP-MutU136G, respectively) showed that the mutation that disrupted the Y-shaped structure (C132U) reduced GFP expression levels to that found with pEAQexpress-HT-GFP-Del-234, while the mutation which maintained it (U136G) had little effect (Figure 4b). These results indicate that an intact Y-shaped secondary structure is, indeed, critical for maintaining high GFP expression levels.

### Complementarity between the 5′ and 3′ UTRs of CPMV RNA-2 is not required for enhanced expression

Nucleotides 134–140 (GUUUAGC), in the minor loop of the predicted Y-shaped structure of the 3′UTR, are complementary to nucleotides 136–142 (CAAAUCG) of the 5′UTR. As regions of complementarity between the 5′ and 3′ UTRs have been shown to be important in controlling expression levels in several plant viral RNAs (Fabian and White, 2004; Guo *et al.*, 2001), the importance of maintaining this complementarity to achieve high levels of expression from the CPMV-*HT* system was investigated. To this end, the seven-nucleotide sequence in the 3′ UTR was either deleted (pEAQexpress-HT-GFP-Del7) or three point mutations were introduced to change the sequence to G**G**U**G**AG**A** (changes shown in bold), thereby destroying its complementarity with the corresponding sequence in the 5′ UTR (pEAQexpress-HT-GFP-3Mut). In both cases, this resulted in at least a threefold decrease in the GFP expression level (Figure 4b). However, the introduction of compensatory mutations in the 5′ UTR of pEAQexpress-HT-GFP-3Mut (to give the sequence C**C**A**C**UC**U,** thereby restoring complementarity between the UTRs in pEAQexpress-HT-GFP-3Mut-Restored) did not restore GFP expression (Figure 4b). Moreover, introduction of the same three point mutations into the 5′UTR of pEAQexpress-HT-GFP (encoding the native 3′UTR) to generate pEAQexpress-HT-GFP-3Mut5′UTR did not reduce GFP expression levels (Figure 4b). Taken together, these results indicate that although nucleotides 134–140 in the 3′ UTR play an important role in maintaining expression levels, this does not operate through direct base pairing between 5′ and 3′ UTRs. It is more likely that these nucleotides are critical for maintaining the correct functioning of the Y-shaped structure as both of the above mutations are predicted to destabilize it.

### Replacement of the 3′ UTR of CPMV RNA-2 with 3′ UTRs from other viruses

**Figure 5:**
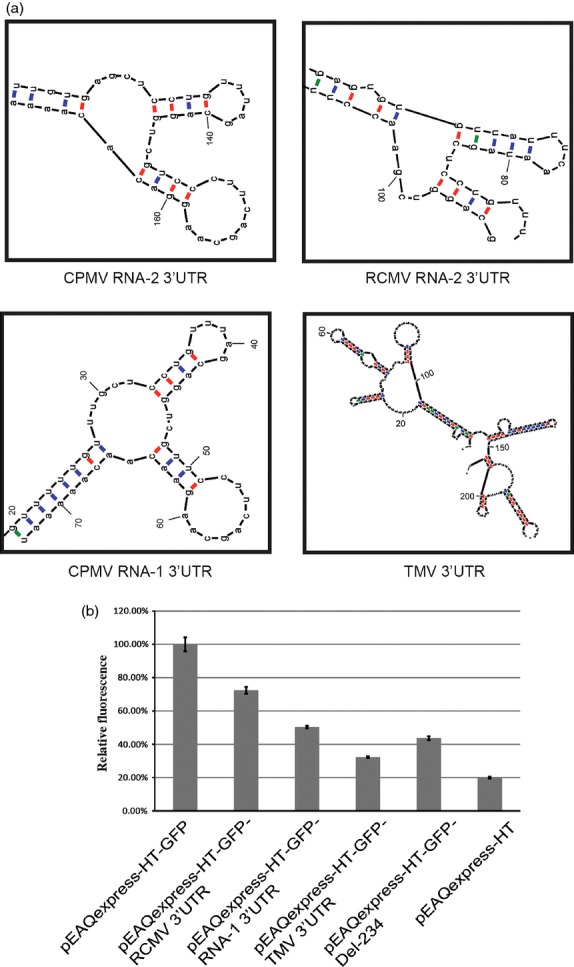
Effect of 3′ UTR substitutions. (a) Secondary structures predicted for 3′ UTRs of RCMV RNA-2, CPMV RNA-1 and TMV and RCMV RNA-2. (b) Relative GFP expression levels based on spectrofluorometric analysis of extracts from leaves harvested at 6 dpi. Values indicate relative expression levels averaged from three technical replicates ± standard error.

If it is the Y-structure rather than the linear sequence of the 3′ UTR of CPMV RNA-2 that is important for enhancing expression, other sequences forming such a structure should give a similar effect. To investigate this possibility, the CPMV RNA-2 3′ UTR was replaced with the either (i) the 3′ UTR of RNA-2 of another comovirus, Red clover mottle virus (RCMV); (ii) the 3′ UTR of CPMV RNA-1 or (iii) the 3′ UTR of an unrelated virus, Tobacco mosaic virus (TMV) strain IM (Figure 5a). The 3′ UTR from RNA-2 of RCMV is longer than that of CPMV RNA-2 (262 versus 183 nucleotides) and has an overall sequence identity of only 32%; however, it contains a region in which 44 of 48 nucleotides are identical to nucleotides 127–175 of the CPMV RNA-2 sequence and which can be folded into a similar Y-shaped structure, with the four differences making compensating changes in one of the stems. The 3′ UTR of CPMV RNA-1 is considerably shorter than its RNA-2 equivalent (82 versus 183 nucleotides) but has a similar Y-shaped structure near its 3′ end (Figure 5a). By contrast, the TMV-IM 3′ UTR is comparable in length (203 versus 183 nucleotides) but is predicted to form a different secondary structure, devoid of any Y-shaped structures similar to those predicted for CPMV RNA-2 but containing a series of pseudo-knots (Figure 5a; van Belkum *et al.*, 1985). To analyse the effects of these alternative 3′UTRs on GFP expression levels, synthetic sequences corresponding to them were inserted into *StuI*-digested pEAQexpress-HT-GFP-Del-234 to create pEAQexpress-HT-GFP-RCMV 3′UTR, pEAQexpress-HT-GFP- RNA-1 3′UTR and pEAQexpress-HT-GFP- TMV 3′UTR, and the levels of GFP expression obtained from these vectors were determined. The presence of the 3′UTR from RNA-2 of RCMV conferred expression levels approximately 75% achieved using the native 3′UTR from CPMV RNA-2 (Figure 5b), while the shorter 3′UTR of CPMV RNA-1gave only half the level. The 3′UTR from TMV was the least effective out of those tested in this experiment, giving expression levels even lower than that seen in the absence of any virus-specific 3′ UTR (Figure 5b). These results are consistent with the requirement for the Y-shaped structure positioned a minimum distance from the end of the structural gene, as suggested by deletion analysis, to achieve maximum enhancement of expression levels.

### The Y-shaped structure enhances mRNA accumulation

**Figure 6:**
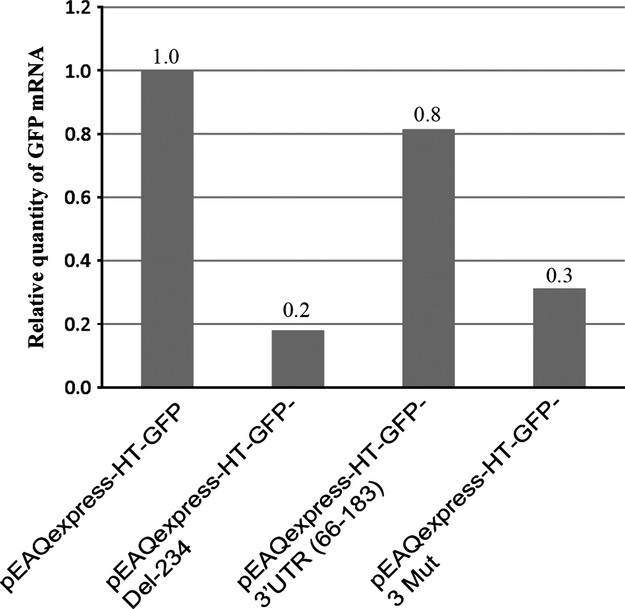
Quantitation of GFP mRNA in plant tissue. Relative levels of mRNA of GFP in leaf tissue infiltrated with four different constructs, as determined by real-time qRT-PCR.

The difference in expression levels of GFP observed for vectors with altered 3′ UTRs could stem either from different translational efficiencies of the GFP mRNAs or, alternatively, from differences in the relative accumulation of the mRNAs. To distinguish between these two possibilities, real-time quantitative reverse transcription (qRT)-PCR was performed to compare relative levels of GFP mRNA in leaf tissue infiltrated with different constructs, two of which [pEAQexpress-HT-GFP and pEAQexpress-HT-GFP-3′UTR(66-183)] were known to give high levels of GFP expression and possess the Y-shaped structure in their 3′ UTR and two which gave low levels of GFP expression and lacked the Y-shaped structure (pEAQexpress-HT-GFP-Del-234 and pEAQexpress-HT-GFP-3Mut). The results show a strong correlation between mRNA levels and GFP expression (Figure 6), suggesting that the higher levels of expression are due to the ability of the Y-shaped structure to enhance mRNA accumulation, rather than any direct effect on translational efficiency. This contrasts with the situation as previously found to be the case for the 5′ UTR of CPMV RNA-2 where enhancement of expression does not correlated with mRNA levels (Sainsbury and Lomonossoff, 2008).

### Creation of expression cassettes of varying strength

**Figure 7:**
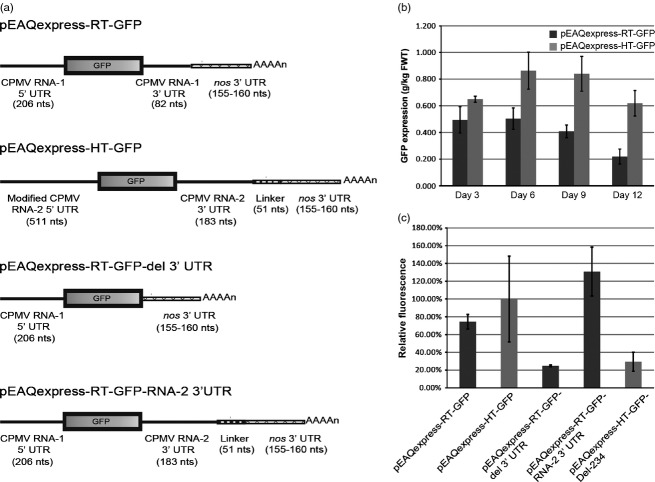
Orthogonal effects of 5′ and 3′ UTRs. (a) Schematic representation of the expression cassettes of the CPMV-RT and CPMV-HT vectors. (b) Expression levels of GFP in the *RT* system (black bars) and the *HT* system (grey bars) based on spectrofluorometric analysis of extracts from leaves harvested on 3, 6, 9 and 12 dpi. (c) Relative GFP expression levels based on spectrofluorometric analysis of extracts from leaves harvested on 6 dpi. Values indicate average expression levels from three biological replicates ± standard error.

The above data indicated that the 5′ and 3′ UTRs of CPMV RNA-2 enhance expression independently through different mechanisms. Hence, it was envisaged that it should be possible to vary the effects of the 5′ and 3′ UTRs orthogonally to create a series of cassettes of varying translational strengths. To investigate this possibility, an expression system similar in design to CPMV-*HT* but utilizing the UTRs from CPMV RNA-1 was created. Briefly, a synthetic cassette consisting of the CaMV 35S promoter, the 5′ UTR of CPMV RNA-1, a polylinker, the 3′ UTR of CPMV RNA-1 and the *nos* terminator was inserted into the T-DNA region of pEAQexpress (Sainsbury *et al.*, 2009) to generate pEAQexpress-RT (Figure S2). The gene encoding GFP was inserted into the polylinker to generate plasmid pEAQexpress-RT-GFP, anticipated to be transcribed to give a mRNA in which the sequence encoding GFP is flanked by the 5′UTR of RNA-1 (206 nucleotides) and a 3′ UTR consisting of the 3′UTR of RNA-1 (82 nucleotides) and the *nos* 3′ UTR (155–160 nucleotides; Figure 7a).

The kinetics of GFP expression obtained with pEAQexpress-RT-GFP were compared with those obtained with pEAQexpress-HT-GFP by preparing protein extracts at various times after infiltration and calculating the amount of GFP per Kg fresh weight of leaf tissue. The results showed that expression from HT vectors was always higher than that obtained from RT vectors and persisted for longer; however, this difference was at its lowest at the earliest time point examined (3 dpi; Figure 7b). The different kinetics of expression are suggestive of differences in mRNA accumulation, rather than translatability, a factor shown to be controlled by the 3′UTR in the case of CPMV-HT. To investigate this possibility, the 3′UTR in pEAQexpress-RT-GFP was either deleted (to give pEAQexpress-RT-GFP-del 3′ UTR) or replaced with the 3′ UTR of RNA-2 (to generate pEAQexpress-RT-GFP-RNA-2 3′UTR), and the GFP expression levels of these constructs were compared with those obtained with pEAQexpress-RT-GFP, pEAQexpress-HT-GFP and pEAQexpress-HT-GFP-Del-234.

As found previously with the HT system, deletion of the entire 3′ UTR from pEAQexpress-RT-GFP resulted in at least a threefold reduction in GFP expression (Figure 7c). On the other hand, insertion of the 3′ UTR of RNA-2 in place of that of RNA-1 resulted in a level of GFP expression higher than that obtained with pEAQexpress-RT-GFP (Figure 7c), a result consistent with the earlier observation that the 3′ UTR of RNA-2 is more effective than that of RNA-1 at enhancing expression. These results show that the levels and duration of foreign gene expression can be modulated by switching 5′ and 3′ UTRs.

## Discussion

The results presented here show, for the first time, that the 3′UTR of RNA-2 of CPMV plays an important role in ensuring high-level expression from the CPMV-*HT* system and its associated pEAQ plasmids through stabilizing the transcribed mRNA. The most important region of the RNA-2 3′UTR in terms of this function is the Y-shaped structure formed by nucleotides 125–165 of the 3′ UTR, and any mutations that delete or disrupt this structure are equivalent to deletion of the entire 3′ UTR. Deletions between the GFP termination codon and the Y-shaped structure have less drastic effects, although removal of 107 nucleotides from this region, as seen in case of pEAQexpress-HT-GFP-3′UTR(108-184), reduces expression to 80% of that achieved with the native 3′ UTR.

The CPMV RNA-2 3′UTR can be partially substituted with that of RCMV RNA-2, a sequence which is predicted to fold into an identical Y-shaped structure. The much shorter 3′ UTR of CPMV RNA-1, despite possessing the Y-shaped structure, is considerably less effective, indicating that the Y-shaped structure must be positioned a minimum distance from the termination codon to operate most efficiently, a result supported by the deletion analysis.

Site-directed mutagenesis experiments provided no evidence that direct interactions between the 5′ and 3′ UTRs are required for high-level expression from CPMV-HT cassettes but, rather, suggested that the two UTRs exert their effects independently. In contrast to the 5′UTR where translation can be modulated without affecting mRNA levels (Sainsbury and Lomonossoff, 2008), deletion of the 3′ UTR dramatically reduces mRNA accumulation. This ability to enhance accumulation suggests that the RNA-2 3′ UTR, and the Y-shaped structure in particular, either improves mRNA stability or enhances transcription. Previous experiments have shown that mutants that disrupt the Y-shaped structure adversely affect RNA-2 accumulation during CPMV replication in protoplasts (Rohll *et al.*, 1993); however, whether this was due to reduced replication, reduced RNA stability or a combination of the two was not addressed. The ability of a plant viral 3′ UTR to stabilize an RNA sequence has previously been reported in the case of TMV where the UTR was able to stabilize RNA molecules in protoplasts, although only when present at the very 3′ end (Gallie and Walbot, 1990). However, when positioned between the sequence of GFP and the *nos* 3′UTR in construct pEAQexpress-HT-GFP-TMV 3′UTR, it proved ineffective, suggesting that the 3′UTR of CPMV RNA-2 and that of TMV stabilize RNA molecules through different mechanisms. Our current data do not permit us to distinguish whether the 3′ UTR enhances mRNA accumulation via increased mRNA stability or enhanced transcription.

A question arising from the current study is its relevance to the mechanism of translation of native CPMV RNA molecules. The transcripts generated during *Agrobacterium*-mediated transient expression from the pEAQ vectors will be capped at the 5′ termini and will contain the sequence of the *nos* 3′UTR, including a poly(A) tract, downstream of the CPMV-specific 3′ UTR. By contrast, natural CPMV RNAs have a small protein (VPg) at their 5′ termini, which is not removed prior to translation (de Varennes *et al.*, 1986) and terminate in a poly(A) tract immediately after the 3′ UTR. Thus, for example, the failure to find evidence for interactions between regions of complementarity in the 5′ and 3′ UTRs may simply reflect the fact that an artificial, cap-dependent mechanism for translation is being used. On the other hand, it should be noted that agro-infiltration of full-length cDNA copies of the CPMV RNAs inserted between the CaMV 35S promoter and the *nos* terminator is a highly efficient means of initiating infection (Liu and Lomonossoff, 2002), implying that the transcribed RNA is biologically active.

Given the independent activities of the 5′ and 3′UTRs in promoting expression, it is now possible to create a range of translational cassettes with different expression properties by ‘mixing and matching’ different UTRs. By varying the efficiency of translation (by mutations within the 5′ UTR) and the level of an mRNA (by altering the 3′ UTR) orthogonally, it is possible to vary both the amount and duration of expression of a heterologous gene. The ability to control the expression levels of different genes expressed within the same cell has immense potential in the field of synthetic biology, for instance in cases where differential enzymes levels are required in a metabolic pathway.

## Experimental procedures

### Design and construction of vectors

pEAQexpress-HT-GFP (Figure 1a; Sainsbury *et al.*, 2009) was used in experiments to investigate the role of the native 3′UTR of CPMV RNA-2. To create constructs lacking either the 3′UTR of RNA-2 plus the 51-nt linker or just the 3′UTR, the region between the unique *Cla*I and *Stu*I sites in the T-DNA region of pEAQexpress-HT-GFP was amplified by PCR using primers P1 and either P2 or P3 (Table S1). This resulted in the generation of fragments encompassing the sequence from the *ClaI* at the start of the *p19* gene to either the beginning of the *nos* terminator or the beginning of the 51-nt linker with *Stu*I sites immediately upstream (Figure S2). Substitution of these fragments, after digestion with *Stu*I and *Cla*I, into similarly digested pEAQexpress-HT-GFP gave constructs pEAQexpress-HT-GFP-Del-234 and pEAQexpress-HT-GFP-Del-183, respectively.

To re-insert the 234-nucleotide sequence encompassing the CPMV RNA-2 3′UTR plus the 51-nucleotide linker sequence into pEAQexpress-HT-GFP-Del-234, a PCR fragment containing this region was amplified from pEAQexpress-HT-GFP using primers P3 and P4, which introduced *Stu*I sites at each end of the amplified fragment. The amplified fragment was digested with *Stu*I and cloned in both orientations into the *Stu*I site of pEAQexpress-HT-GFP-Del-234 to give pEAQexpress-HT-GFP-Del-234-corr 234 and pEAQexpress-HT-GFP-Del-234-opp 234. To re-insert just the 183 nucleotides corresponding to the CPMV RNA-2 3′UTR, this region was amplified using primers P6 and P4 and cloned to generate pEAQexpress-HT-GFP-Del-234-corr 183 (Figure 2a).

To create deletions within the CPMV RNA-2 3′UTR, the region between the *Cla*I and *Stu*I sites of pEAQexpress-HT-GFP was amplified using primer combinations P1 and P7 to obtain construct pEAQexpress-HT-GFP-3′UTR(66-183), P1 and P8 to obtain pEAQexpress-HT-GFP-3′UTR(108-183), and P1 and P9 to obtain pEAQexpress-HT-GFP-3′UTR(142-183). These fragments were ligated into *Cla*I*-Stu*I-digested pEAQexpress-HT-GFP. To create pEAQexpress-HT-GFP-3′UTR(1-141), a fragment of the vector pEAQexpress-HT-GFP was amplified by PCR using primers P11 and P10, digested with *Stu*I and ligated into *Stu*I*-*digested pEAQexpress-HT-GFP-Del-234.

The cDNA sequences of 3′UTRs of RCMV RNA-2 (accession number NC_003738.1), CPMV RNA-1 (accession number NC_003549) and TMV (accession number AB369276.1) were obtained from GenBank®. DNA was synthesized by GENEART® (Life Technologies, Grand Island, NY) and cloned into *Stu*I-digested construct pEAQexpress-HT-GFP-Del-234.

A cDNA comprising the 5′ UTR of RNA-1 (206 bp), a polylinker (52 bp) and the 3′ UTR of RNA-1 (82 bp) (Figure S2) was cloned into pEAQexpress (Sainsbury *et al.*, 2009) to create construct pEAQexpress-*RT*. The gene encoding GFP was cloned into the *Xho*I and *Xma*I sites of the polylinker to generate pEAQexpress-*RT*-GFP, in which *gfp* is flanked by the 5′ UTR and the 3′ UTR of CPMV RNA-1 and is under the control of a 35S promoter and a *nos* terminator.

pEAQexpress-RT-GFP-del3′UTR was created by amplifying the 1.5-kb region immediately downstream of the 3′UTR of pEAQexpress-RT-GFP using primers P26 and P27 and inserting the amplified fragment upstream of the 3′UTR using sites *Xma*I and *Bam*HI, thereby deleting the entire 3′UTR. Similarly, pEAQexpress-RT-GFP-RNA-2 3′UTR was created by amplifying a 1.8-kb region from pEAQ-HT (Sainsbury *et al.*, 2009) consisting of the 3′UTR of RNA-2 and DNA downstream of it (using P26 and P28) and inserting the amplified product upstream of the 3′UTR of pEAQexpress-RT-GFP, thereby replacing the 3′UTR of RNA-1 with that of RNA-2.

### Site-directed mutagenesis

Point mutations were introduced into 5′ and 3′ UTR regions of pEAQexpress-HT-GFP using the QUIKCHANGE® (Agilent Technologies Inc. Santa Clara, CA) method according to the instructions provided by the manufacturer (Agilent technologies). Site-directed primers were designed using the Web program provided by the manufacturer.

### RNA secondary structure prediction

RNA secondary structures mentioned in this study were predicted using Mfold Web Server, available from http://mfold.rna.albany.edu/?q=mfold/RNA-Folding-Form (Zuker, 2003).

### Agro-infiltration

Binary plasmids were maintained in *Agrobacterium tumefaciens* strain LBA4404, which was transformed by electroporation and grown on LB containing 50 μg/mL rifampicin and 50 μg/mL kanamycin. Stationary-phase liquid cultures were pelleted by centrifugation at 2000 ***g*** for 20 min and resuspended in MMA <10 mm MES [2-(N-morpholino)ethanesulfonic acid] at pH 5.6, 10 mm MgCl_2_, 100 μm acetosyringone (4-hydroxy-3,5-dimethoxyacetophenone)} to make a solution of final optical density at 600 nm (OD_600_) = 0.4. The suspensions were left at room temperature for 0.5–3 h prior to infiltration to allow acetosyringone in the buffer to induce virulence of agrobacteria. The agro-suspension was pressure-infiltrated into young fully expanded leaves of 3- to 4-week-old *N. benthamiana* plants with the help of a needle-less syringe. Infiltrated leaves were harvested at various time points, up to 12 days postinfiltration.

### Preparation of total protein extract

For each construct used, 4–6 leaf discs were generated from infiltrated leaves using a sterile cork borer. The leaf discs were homogenized in three volumes of extraction buffer [50 mm Tris–HCl pH 7.25, 150 mm NaCl, 2 mm ethylenediaminetetraacetic acid (EDTA)] using ceramic beads in THERMO® Savant FastPrep FP120 Homogenizer (Thermo Fischer Scientific, Loughborough, UK). The homogenate was clarified by centrifugation for 15 min at 16 000 ***g*** using a cold bench-top centrifuge. The supernatant represented the total soluble protein extract.

### GFP fluorescence assay

GFP fluorescence measurements were made using a protocol modified from Richards *et al*. (2003). Soluble protein extracts were diluted in the ratio 1:100 in 0.1 m Na_2_CO_3_ and loaded in triplicate onto a fluorescently neutral black COSTAR® 96-well plate (Sigma-Aldrich, Suffolk, UK). Recombinant GFP (Clontech, Saint-Germain-en-Laye, France), which is the same variant of GFP as expressed by the pEAQ vectors, was diluted in plant extract to generate standard curves. Excitation (wavelength of 395 nm) and emission (509 nm) maxima were matched to Clontech's GFP and read using a SPECTRAmax spectrofluorometer (Molecular Devices, Sunnyvale, CA). Following subtraction of the signal from a control extract of non-infiltrated leaves, values representing GFP fluorescence were entered into the linear regression for the standard curve to calculate the amount of GFP present in each sample. To calculate relative expression levels for all constructs, expression achieved from construct pEAQexpress-HT-GFP was set to 100%, and expression from other constructs was calculated as a percentage of the expression from pEAQexpression-HT-GFP. Measurements were made in triplicate to account for experimental variation and averaged to give a final value for each construct. In the case of time-course experiments, the average from three biological replicates is reported. Error bars denote standard error of the mean of the three (technical or biological) replicates with a 95% confidence interval. This was calculated using the formula: 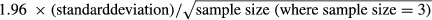.

### Real-time quantitative RT-PCR

Total RNA from agro-infiltrated *N. benthamiana* leaves was isolated using RNeasy® Plant Mini Kit (QIAGEN®, Manchester, UK). After treatment with DNaseI, 2 μg of RNA was used for reverse transcription with oligo(dT) primers using ProtoScript® m-MuLV First Strand cDNA Synthesis Kit (New England BioLabs, Hertfordshire, UK). Quantitative RT-PCR was performed using SYBR®Green JumpStart™ Taq ReadyMix™ (Sigma-Aldrich) with primers P22 and P23 (to detect GFP transcripts) and primers P24 and P25 (to detect β-actin transcripts) in the Chromo4 Q-PCR Opticon cycler (Bio-Rad, Hercules, CA). Relative RNA amounts were determined by the ΔΔCt method using β-actin mRNA for normalization (Livak and Schmittgen, 2001).

### Visualization and Photography

GFP expression in infiltrated *N. benthamiana* plants was monitored with a 100 AP handheld UV lamp (Blak Ray®, Upland, CA). A Nikon® D700 (Nikon, Tokyo, Japan) digital camera with a 60-mm macro lens was used for image acquisition under visible light or, for the detection of GFP, under UV illumination. Images were edited using Photoshop CS4 and Illustrator (Adobe, San Jose, CA).

## Supporting Information

Figure S1 Schematic diagram depicting the entire 3′ UTR of pEAQexpress-HT-GFP and its corresponding sequence. CPMV-specific sequences are in bold.

Figure S2 Schematic diagram depicting the region of study of pEAQexpress-RT. CPMV-specific sequences are in bold.

Table S1 Table of primers used in this study.

## References

[b1] van Belkum A, Abrahams JP, Pleij CWA, Bosch L (1985). Five pseudoknots are present at the 204 nucleotide long 3′ noncoding region of tobacco mosaic virus RNA. Nucleic Acids Res.

[b2] Bevan M, Barnes WM, Chilton MD (1983). Structure and transcription of the nopaline synthase gene region of T-DNA. Nucleic Acids Res.

[b3] Cañizares MC, Liu L, Perrin Y, Tsakiris E, Lomonossoff GP (2006). A bipartite system for the constitutive and inducible expression of high levels of foreign proteins in plants. Plant Biotechnol. J.

[b4] Eggen R, Verver J, Wellink J, Pleij K, van Kammen A, Goldbach R (1989). Analysis of sequences involved in cowpea mosaic virus RNA replication using site-specific mutants. Virology.

[b5] van Engelen FA, Molthoff JW, Conner AJ, Nap JP, Pereira A, Stiekema WJ (1995). pBINPLUS – an improved plant transformation vector based on pBIN19. Transgenic Res.

[b6] Fabian MR, White KA (2004). 5′-3′ RNA-RNA interaction facilitates cap- and poly (A) tail-independent translation of Tomato bushy stunt virus mRNA. J. Biol. Chem.

[b7] Gallie DR, Walbot V (1990). RNA pseudoknot domain of tobacco mosaic virus can functionally substitute for a poly(A) tail in plant and animal cells. Genes Dev.

[b8] Guo L, Allen EM, Miller WA (2001). Base-pairing between untranslated regions facilitates translation of uncapped, nonpolyadenylated viral RNA. Mol. Cell.

[b9] Liu L, Lomonossoff GP (2002). Agroinfection as a rapid method for propagating Cowpea mosaic virus-based constructs. J. Virol. Methods.

[b10] Livak KJ, Schmittgen TD (2001). Analysis of relative gene expression data using real- time quantitative PCR and the *2*^*−ΔΔCT*^ Method. Methods.

[b11] Miller WA, Wang Z, Trader K (2007). The amazing diversity of cap –independent translation elements in the 3′-untranslated regions of plant viral RNAs. Biochem. Soc. Trans.

[b12] Peyret H, Lomonossoff GP (2013). The pEAQ vector series: the easy and quick way to produce recombinant proteins in plants. Plant Mol. Biol.

[b13] Richards HA, Halfhill MD, Millwood RJ, Stewart CN (2003). Quantitative GFP fluorescence as an indicator of recombinant protein synthesis in transgenic plants. Plant Cell Rep.

[b14] Rohll JB, Holness CL, Lomonossoff GP, Maule AJ (1993). 3′-terminal nucleotide sequences important for the accumulation of cowpea mosaic virus M-RNA. Virology.

[b15] Sainsbury F, Lomonossoff GP (2008). Extremely high-level and rapid transient protein production in plants without the use of viral replication. Plant Physiol.

[b16] Sainsbury F, Thuenemann EC, Lomonossoff GP (2009). pEAQ: versatile expression vectors for easy and quick transient expression of heterologous proteins in plants. Plant Biotechnol. J.

[b17] Sainsbury F, Sack M, Stadlmann J, Quendler H, Fisher R, Lomonossoff GP (2010). Rapid transient production in plants by replicating and non-replicating vectors yields high quality functional anti-HIV antibody. PLoS ONE.

[b18] Thuenemann EC, Meyers AE, Verwey J, Rybicki EP, Lomonossoff GP (2013). A method for rapid production of heteromultimeric protein complexes in plants: assembly of protective bluetongue virus-like particles. Plant Biotechnol. J.

[b19] de Varennes A, Lomonossoff GP, Shanks M, Maule AJ (1986). The stability of cowpea mosaic virus VPg in reticulocyte lysates. J. Gen. Virol.

[b20] Zuker M (2003). Mfold web server for nucleic acid folding and hybridization prediction. Nucleic Acids Res.

