# Supplementary figures and images for: Fine-tuning levels of heterologous gene expression in plants by orthogonal variation of the untranslated regions of a nonreplicating transient expression system
Source: Plant Biotechnol J. 2014 Mar 12;12(6):718–27. doi: 10.1111/pbi.12175 (PMC4265252; doi:10.1111/pbi.12175)

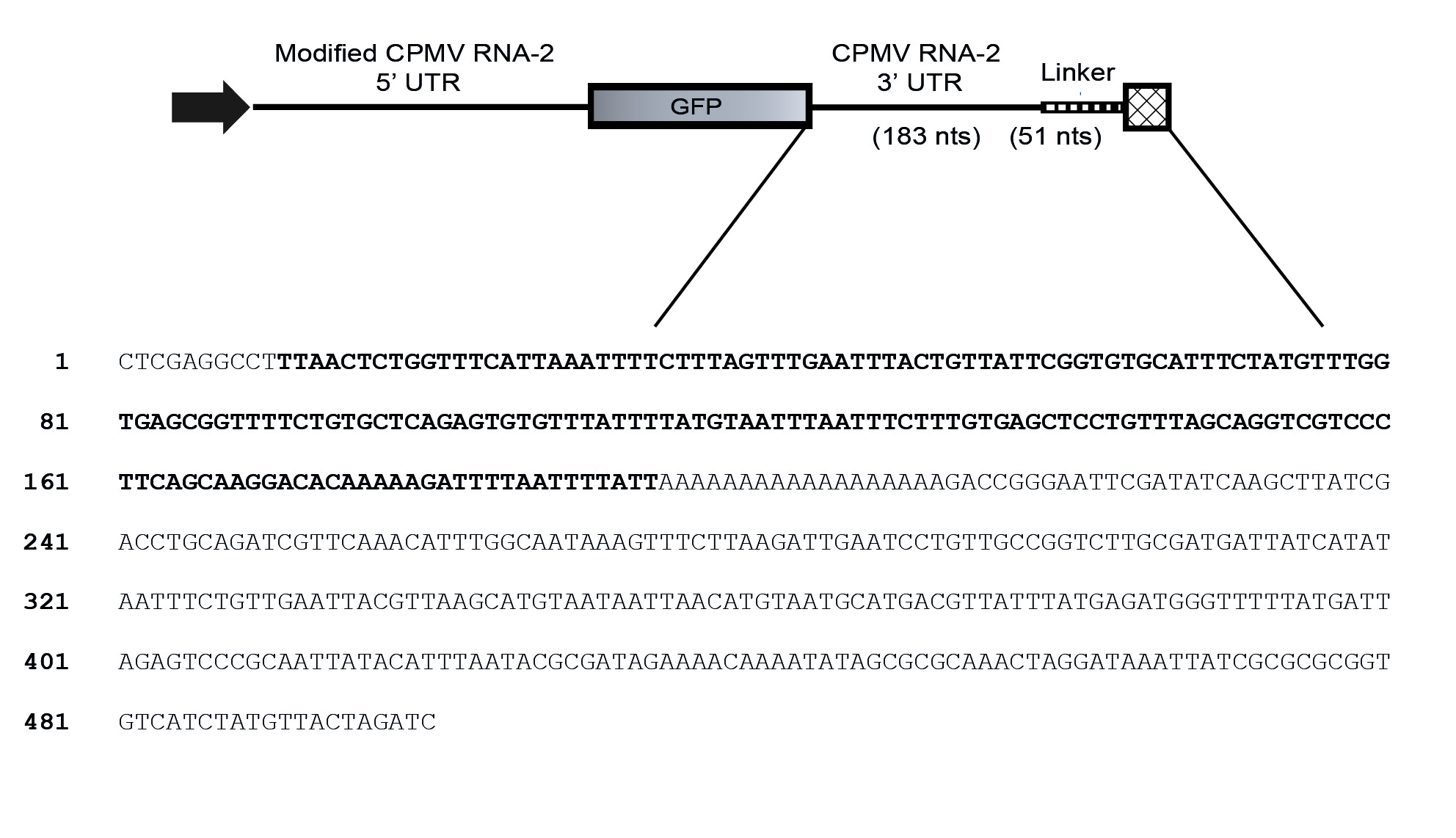

Supplement: Figure S1 — Schematic diagram depicting the entire 3′ UTR of pEAQexpress-HT-GFP and its corresponding sequence. CPMV-specific sequences are in bold. [file pbi0012-0718-SD1.jpg]

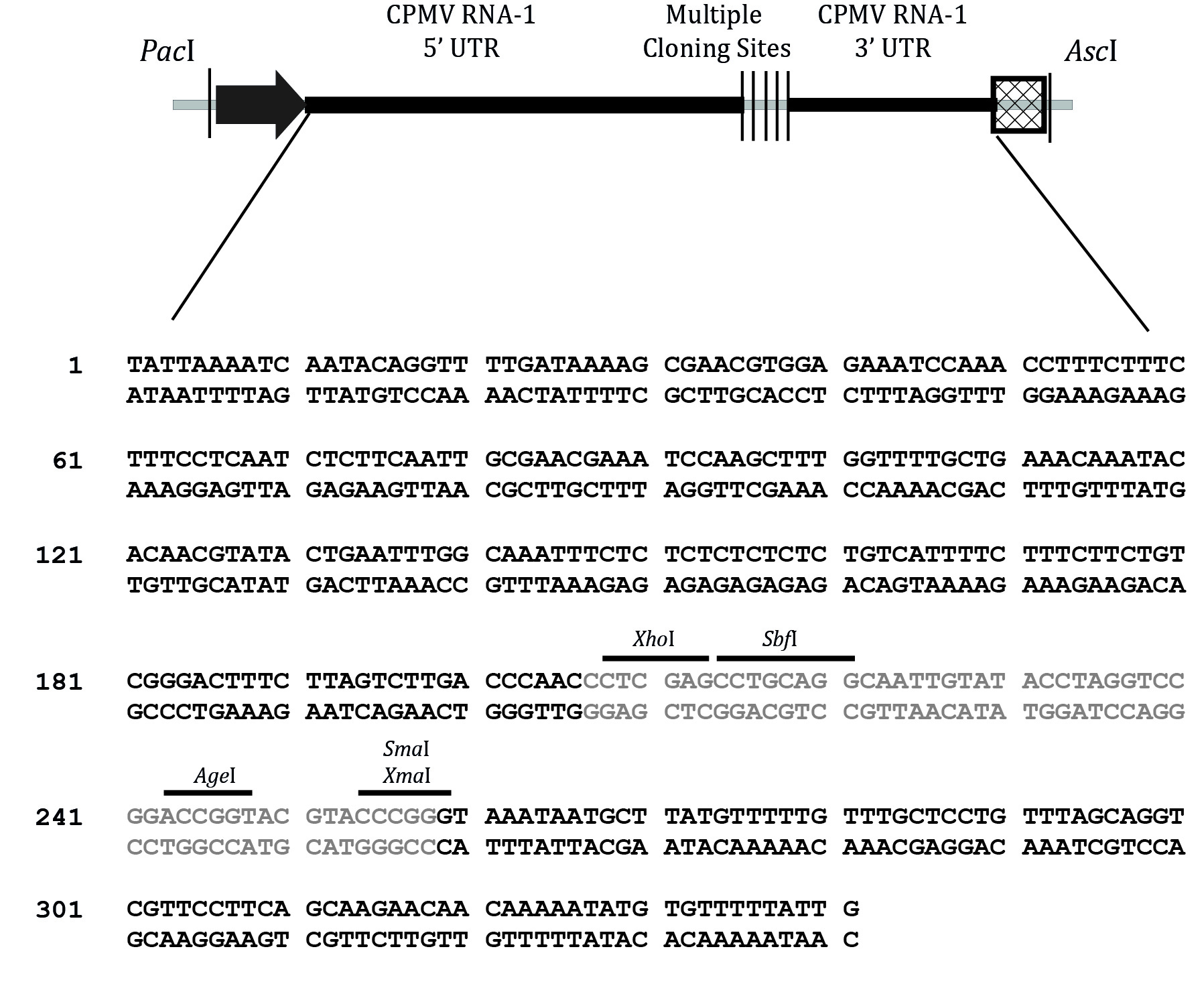

Supplement: Figure S2 — Schematic diagram depicting the region of study of pEAQexpress-RT. CPMV-specific sequences are in bold. [file pbi0012-0718-SD2.jpg]
