# Supplementary material for: Fine-tuning levels of heterologous gene expression in plants by orthogonal variation of the untranslated regions of a nonreplicating transient expression system
Source: Plant Biotechnol J. 2014 Mar 12;12(6):718–27. doi: 10.1111/pbi.12175 (PMC4265252; doi:10.1111/pbi.12175)
Supplement: Table S1 — Table of primers used in this study. [file pbi0012-0718-SD3.docx]

**Table S1:** Table of primers used in this study

Oligos used for PCR amplification and mutagenesis are listed below. Restriction sites are in upper case.

| **Oligo** | **Sequence** | **Function** |
| --- | --- | --- |
| **P1** | atccccttaa**ATCGAT**atggaac | Sense primer for amplification of the ClaI containing region of  pEAQexpress-HT-GFP |
| **P2** | tac**AGGCCT**gcagatcgttcaaac | Antisense primer for amplification of the StuI containing region of  pEAQexpress-HT-GFP |
| **P3** | tac**AGGCCT**aaaaaaaaaaaaaa | Antisense primer for amplification of the StuI containing region of pEAQexpress-HT-GFP |
| **P4** | atc**AGGCCT**gcaggtcgataagcttgatatc | Sense primer for amplification of the region of pEAQexpress-HT-GFP, containing 3’UTR plus 51 nt linker, with StuI site |
| **P5** | ac**AGGCCT**ttaactctggtttcatta | Antisense primer for amplification of the region of pEAQexpress-HT-GFP, containing 3’UTR plus 51 nt linker, with StuI site |
| **P6** | tac**AGGCCT**aataaaattaaaatctttttgtgtccttgctgaaggggac | Sense primer for amplification of the region of pEAQexpress-HT-GFP, containing 3’UTR without 51 nt linker, with StuI site |
| **P7** | tac**AGGCCT**tggtgagcggttttctg | Antisense primer for deletion of 65 nts from 3’UTR of CPMV RNA-2 with StuI site |
| **P8** | tac**AGGCCT**gtaatttaatttctttgtgagc | Antisense primer for deletion of 107 nt from 3’UTR of CPMV RNA-2 with StuI site |
| **P9** | tac**AGGCCT**ggtcgtcccttca | Antisense primer for deletion of 141 nt from 3’UTR of CPMV RNA-2 with StuI site |
| **P10** | tag**AGGCCT**gctgaagggacgacc | Sense primer to delete 43 nt from 3’end of 3’UTR of CPMV RNA-2 with StuI site |
| **P11** | ac**AGGCCT**ttaactctggtttcatta | Antisense primer to delete 43 nt from 3’end of 3’UTR of CPMV RNA-2 with StuI site |
| **P12** | gggacgacctgctaaacaagagctcacaaagaaa | Sense primer for introduction of point mutation C132U disrupting Y-shaped structure |
| **P13** | tttctttgtgagctcttgtttagcaggtcgtccc | Antisense primer to introduce point mutation C132U disrupting Y-shaped structure |
| **P14** | ctgaagggacgacctgctacacaggagctcac | Sense primer for introduction of the structurally neutral point mutation U136G |
| **P15** | gtgagctcctgtgtagcaggtcgtcccttcag | Antisense primer for introduction of the structurally neutral point mutation U136G |
| **P16** | ctttgtgagctcctaggtcgtcccttcagcaa | Sense primer to delete GUUUAGC motif from 3’UTR of CPMV RNA-2 |
| **P17** | ttgctgaagggacgacctaggagctcacaaag | Antisense primer to delete GUUUAGC motif from 3’UTR of CPMV RNA-2 |
| **P18** | ccttgctgaagggacgaccttctcaccaggagctcacaaagaaatt | Sense primer to change GUUUAGC motif in the Y-shaped structure to GGUGAGA |
| **P19** | aatttctttgtgagctcctggtgagaaggtcgtcccttcagcaagg | Antisense primer to change GUUUAGC motif in the Y-shaped structure to GGUGAGA |
| **P20** | ggccgaagccagagtggacaacgttgaag | Sense primer to change CAAAUCG motif in the 5’UTR of CPMV RNA-2 to CCACUCU |
| **P21** | cttcaacgttgtccactctggcttcggcc | Antisense primer to change CAAAUCG motif in the 5’UTR of CPMV RNA-2 to CCACUCU |
| **P22** | cttgacttcagcacgtgtcttgtagttccc | Sense primer for qPCR to detect GFP transcript |
| **P23** | agagggtgaaggtgatgcaacatacgg | Antisense primer for qPCR to detect GFP transcript |
| **P24** | cagaaagaggctactcttttaccaccacgg | Sense primer for qPCR to detect β-actin transcripts |
| **P25** | gtggtttcatgaatgccagcagcttcc | Antisense primer for qPCR to detect β-actin transcripts |
| **P26** | cgat**CCCGGG**ttgatcgttcaaacatttgg | Sense primer to amplify the region downstream of the *nos* terminator in pEAQexpress-RT |
| **P27** | gactctaga **GGATCC** ccttaaatcgatatgg | Antisense primer for amplification of the region downstream of the P19 gene in pEAQexpress-RT |
| **P28** | cgat**CCCGGG**cttaactctggtttcattaa | Sense primer for amplification of the region downstream (and including) the 3’ UTR of RNA-2 in pEAQexpress-HT-GFP |
